# Associations Between Online Search Trends and Outpatient Visits for Common Medical Symptoms in the United States from 2004 to 2019: Time Series Ecological Study

**DOI:** 10.2196/77274

**Published:** 2025-11-04

**Authors:** Ryan Heumann, Steven R Steinhubl

**Affiliations:** 1 Indiana University School of Medicine, 340 W 10th St, Indianapolis, IN, 46202, United States, 1 317-274-8157; 2 Weldon School of Biomedical Engineering, Purdue University West Lafayette, West Lafayette, IN, United States

**Keywords:** internet, health information, patient education, ambulatory care, primary care, infodemiology, digital health

## Abstract

**Background:**

The seemingly endless amount of information available on the internet at the touch of a few buttons has increasingly served as a resource for individuals to find health information over the last 20+ years. Previous research in the field of infodemiology suggests that this change in use of the internet by the public to find health information has an impact on their interaction with the health care system. However, to this date, no study has directly juxtaposed the growth of internet searching and the number of visits to ambulatory care centers for the same concerns over the last two decades of internet growth.

**Objective:**

This study aimed to explore the relationship between online searches for three common primary care concerns and ambulatory visits for those same concerns during 2004‐2019.

**Methods:**

The National Ambulatory Medical Care Survey and National Hospital Ambulatory Medical Care Survey data were searched between the years 2004 and 2019 in order to estimate the number of annual ambulatory care visits for cough, sore throat, and abdominal pain. Google Trends data were explored to analyze the number of people searching for information about the same complaints over the same years. Cross correlation, time series regression, and Granger causality analysis were performed to analyze patterns and causality.

**Results:**

Google Trends data showed that the number of searches for the common primary care symptoms “cough,” “sore throat,” and “stomach pain” in the United States grew by 208%, 290%, and 490%, respectively, between 2004 and 2019. However, over the same time, United States population-adjusted outpatient visits for cough and sore throat decreased by 41.5% and 40%, respectively, and stomach pain visits remained unchanged. According to conservative estimation, analysis concluded that an increase in internet searches for cough can predict a decline in ambulatory visits for that symptom.

**Conclusions:**

This supports that, on a population level, exploring online health information about some common, acute symptoms did not lead to increase in care seeking, but instead, suggests that a substantial portion of the population found the information to be reassuring or informative enough to not feel the need to seek care from a primary health care provider. With the rapid evolution and availability of more detailed and personalized information from various large language models, it is likely that internet search habits of users will continue to grow, and with it, continue to transform interactions with the health care system.

## Introduction

The term “infodemiology” was created in 2002 by Gunther Eysenbach in an attempt to describe an approach for revolutionizing public health by using data monitoring and mining [1-3]. It is defined as “the science of distribution and determinants of information in an electronic medium, specifically the internet, or in a population, with the ultimate aim to inform public health and public policy” [3]. The underlying theory is that changes in information and communication patterns may either reflect or cause changes in population health [3]. The field is further divided into supply-based infodemiology, that is the study of the amount and quality of information on the internet, and demand-based infodemiology, that is the study of search patterns in a population [3]. This discussion will center around the latter and its relationship with ambulatory care seeking.

One of the original infodemiology studies, by Eysenbach [3] in 2006, showed that internet searches preceded visits to a primary care physician by one week, suggesting that people often consulted the internet first before seeing a physician. Since then, the number of global internet users and with it, the volume of searches has grown exponentially—from 200 million a day in 2004 to a projected 13.6 billion in 2025—and so has the ability of the internet to affect health care [4,5]. A study by Finney et al [6] found that the number of individuals who had used the internet first in their most recent search for health information grew from 61.2% in 2008, to 69.6% in 2011, and to 74.9% in 2017. Furthermore, the Health Information National Trends Survey (HINTS) in 2022 found that, of respondents who use the internet, 85.3% have used it to look for health information in the past year [7].

What qualifies as health information in this context is not well-defined, in part because the motivations individuals have for using the internet in order to inform a health-affecting decision are varied. While the data are limited, several interview-based studies find that some (~60% according to one study [8]) turn to the internet first to determine whether their symptoms seem serious enough to warrant visiting a doctor, with the hope being that they can find reassurance and save time and money [8,9]. If they fall into the ~40% of people who are typically reassured by an online health information search, the road might end there [10]. However, if they are not reassured, or even fall into the ~30% of people who tend to be more worried after a search, then they typically make an appointment with a physician [9,10]. In this case, a separate motivation to search for information might be to explore possible diagnoses, symptoms, treatments, or better understand medical jargon in order to be best prepared for their visit [9,11]. After a visit, individuals have cited clarifying physician instruction, finding a second opinion, and reading forums of others with their diagnosis as further motivations for searching the internet [9,12]. In fact, in 2022, 54% of survey respondents found internet information helpful to understanding information given to them by a physician [8]. No matter what the motivation, only 8% of people believe that using the internet has an adverse effect on their relationship with their physician and one of every four people think it has a positive impact [13].

It has been theorized that the internet empowers patients by giving them the ability to actively seek information, which improves their agency and future interactions with their health care provider [14]. This sense of empowerment and control has been shown to lead to more discussion with physicians and better ability to elicit information during consultations [14]. This generally leads to better treatment outcomes and higher levels of patient satisfaction. Patient-physician communication can also improve from this re-balanced division of knowledge, which can additionally lead to more recall and understanding of information and improved emotional state [14].

We sought to explore the relationship between the known increase in internet search volume for health information and outpatient care seeking over the last two decades in the United States. To do this, we compared the trajectory in the yearly number of online searches for specific acute symptoms with that of the number of people seeking outpatient care for that same symptom that same year. Though previous research discussed here offers motivations for internet searching as well as occasional estimates of impact on care, none analyze the change in behaviors throughout the evolution of the internet in society. We hypothesized that the increasing use of online health information could potentially impact peoples’ care-seeking behavior in one of several ways.

First, it is possible that as online search volume goes up, so does care seeking. While this could potentially be due to an increase in the prevalence of a symptom over time, we selected three symptoms—cough, sore throat and stomach pain—that despite yearly fluctuations would not be expected to increase on a year-to-year basis. That would leave an alternative explanation for a potential yearly increase in outpatient visits for that symptom, that online information seeking heightened a majority of users’ concerns and prompted them to seek care when they might not otherwise have done so. Second, it is also possible that there could be an inverse relationship between the search volume for a specific symptom and care seeking for that same symptom. This might suggest that the information found online reassured the user enough to decide not to seek care.

## Methods

### United States Outpatient Visit Data

The National Ambulatory Medical Care Survey (NAMCS) and National Hospital Ambulatory Medical Care Survey (NHAMCS) from the Division of Health Care Statistics of the National Center for Health Statistics, Centers for Disease Control and Prevention were used to determine common primary care reasons for visit. These surveys are sent out to a sample of US-based physicians, and responses are used to create country-wide estimations, with standard error reported, in various domains. The NAMCS analyzes office-based health care providers while the NHAMCS analyzes emergency departments (EDs) and hospital-based outpatient centers. Taken together, the aim is to provide a comprehensive picture of ambulatory care use in the United States for any given year [15].

For the purposes of this analysis, data collection was done only until 2019 in order to not analyze data that might have been skewed by factors attributable to the COVID-19 pandemic that began in 2020 such as quarantine and decreased availability of ambulatory care sites. In the NAMCS, the top 20 patient-reported reasons for visit were available for every year from 2000 to 2019 with the exception of 2017. In 2017, a change in collection methods led to difficulty in processing the data, which has indefinitely postponed the release of data [16]. In all 19 years of data available, cough, symptoms referable to the throat, and stomach pain, cramps, and spasms were listed in the top 20 reasons for visit.

The NHAMCS provides separate reports for ED visits and hospital-based outpatient visits. Data for each year from 2000 to 2019 were available for ED visits, but only until 2011 for hospital-based outpatient visits. The hospital-based outpatient data from 2012 to 2017 was not released due to quality assurance issues while the survey was not conducted in 2018‐2019 because of budget constraints [16]. The top 20 patient-reported reasons for visit were collected until 2007 when ED data started only recording the top 10.

### Internet Search Data

To provide data, Google Trends finds the percentage of all searches for a certain time frame that were for the input term. It then scales these percentages to the same time period in which that input term had the highest percentage of searches, giving an array of values from 0 to 100, with 100 being the most popular month and 0 being the least [17]. Google Trends was searched for the terms “cough,” “sore throat,” and “stomach pain” from January 1st, 2004 (the earliest data available) to December 31, 2019 (to eliminate most COVID-19 data) in the United States across all categories. The results were displayed as a graph with relatively popularity of the term available monthly from 2004 to 2019.

A secondary resource, Glimpse, which provides data similar to Google Trends, was used in attempt to convert relative popularity to gross number of individual searches for the same time period. Glimpse was used to find the gross number of searches in the month that was the most popular according to Google Trends (relative popularity of 100). Then Google Trends data was used to calculate the gross number of searches for the rest of the months. Because Glimpse provides very limited information about their methods, graphs were compared to Google Trends data, which provides much more transparency. When comparing relative popularity of search terms reported by Glimpse and Google Trends, the Pearson correlation coefficients were 0.99 for cough, sore throat, and stomach pain, indicating that the data were consistent enough between platforms to justify using Glimpse in a very limited capacity.

To decide the query term analyzed, the goal was to choose a term that a patient would reasonably search when feeling the given symptom. The query “cough” was searched to correspond to cough for obvious reasons. For symptoms referable to the throat, this was taken to mean sore throat, so that was the query analyzed. For stomach pain, cramps, and spasms, the decision was made to choose “stomach pain” because it seemed the most general and colloquial.

### Statistical Analysis

NAMCS and NHAMCS visits were adjusted to estimate the number of visits that would have occurred given the 2019 population in order to more equally compare them with internet search data that is already adjusted for population change. The number of visits according to the NAMCS and NHAMCS data were aggregated for each year to provide one measure of total yearly ambulatory visits. Each month of Google Trends data was aggregated for a given year as well. The result was a list of yearly internet searches and a list of yearly ambulatory visits for cough, sore throat, and abdominal pain for each year from 2004‐2019.

In the cases in which one of the three symptoms of interest were not reported in the top 10 or top 20 symptoms of NHAMCS data, one of two paths was taken. In the first method, maximum estimation, the number of visits for the symptom of interest would be recorded as the number of visits for the least common symptom that was still listed. Because the interest is in analyzing the decreasing trend in visits, using this number, that represents the maximum number of visits the symptom of interest could have been, provides a conservative estimate. The other method consisted of using linear interpolation to estimate the missing data. Analysis was done using both sets of data and the more conservative method was selected in order to prevent spurious conclusions. To determine significance in the change of number of ambulatory visits from 2004 to 2019 for each symptom, a two-tailed *z* test was performed. Standard error was reported along with the visits from the NAMCS and NHAMCS data and was used during analysis.

For both the data using maximum estimation and linear interpolation estimation, stationarity was determined for each symptom for search data and ambulatory visit data. Once stationarity was achieved (using differencing or double differencing), cross correlation, time series regression, and Granger causality tests were performed to analyze the relationship between internet searches and ambulatory visits for each of the three symptoms of interest.

### Ethical Considerations

All information used in this manuscript was publicly available and deidentified. Because we did not perform any human or animal subjects research, ethics board approval was not applied for per the Institutional Review Board of Purdue University.

## Results

### Findings from NAMCS and NHAMCS Data

**Figure 1.**
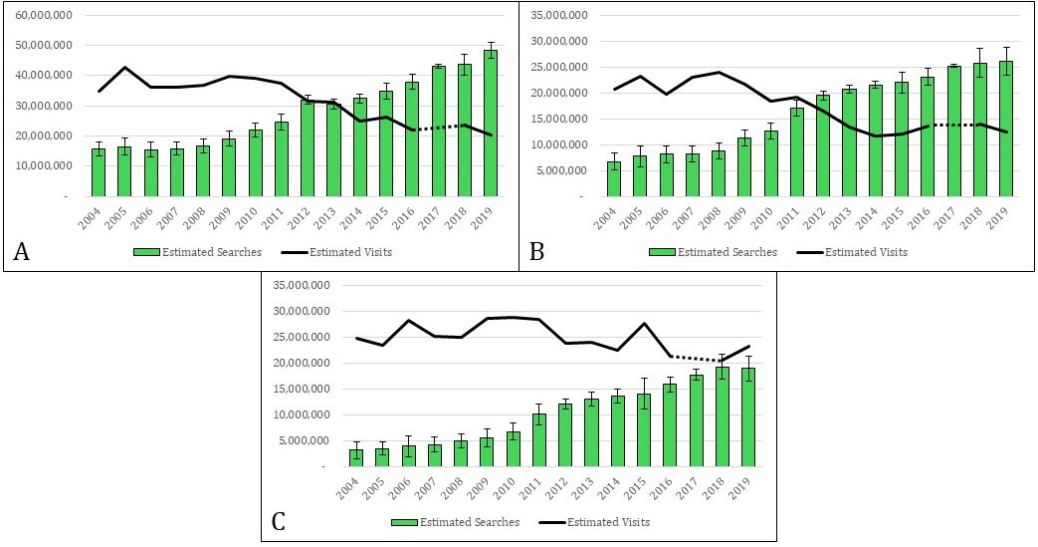
Estimated visits for cough (**A**), sore throat (**B**), and stomach pain (**C**) based on NAMCS/NHAMCS data compared to Google Trends cumulative popularity for the corresponding query terms. NAMCS: National Ambulatory Medical Care Survey; NHAMCS: National Hospital Ambulatory Medical Care Survey.

For cough and sore throat, there was a significant decrease over time in the population-adjusted number of outpatient visits reported by NAMCS and NHAMCS data (Figure 1). Between 2004 and 2019, there was a 41.5% decrease in the number of visits for cough (~35 million to ~20.4 million, 95% CI −22,050,103 to −7,030,485, *P*<.001) and a 40% decrease in the number of visits for sore throat (~20.7 million to ~12.4 million, 95% CI −14,654,695 to −1,921,239, *P*<.01). During this same period, the number of searches reported by Google Trends increased for cough by 208% from ~15.7 million in 2004 to ~48.4 million in 2019 and by 290% for sore throat from ~6.7 million to ~26.2 million. While online searches for stomach pain increased by 490%, from ~3.2 million to ~19 million, visits remained relatively consistent, with a non-significant decrease of 6.3% from ~24.8 million in 2004 to ~23.2 million in 2019 (95% CI −7,494,982 to 4,372,021, *P*=.61; Figure 1).

### Cross Correlation

When using linear interpolation estimation, cross correlation was insignificant for cough, but significant for abdominal pain with an R value of −0.595 (*P*=.025) at a lag of 5 years (the pattern in internet searches followed the pattern of clinical encounters 5 years prior). However, when using maximum estimation, cross correlation became insignificant for all three symptoms (Table 1).

**Table 1.** Summary of cross correlation, time series regression, and Granger causality testing for each method of estimating missing data.

Symptom	Cross correlation	Regression coefficient	Granger causality
Linear interpolation estimation			
Cough	No (*P*=.061)	−0.84 (*P*<.01^a^)	Yes (*P*=.020)^a^
Sore throat	No (*P*=.09)	−1.32 (*P*=.043)^a^	Yes (*P*=.045)^a^
Abdominal pain	Yes (*r*=−.595, *P*=.025)^a^	−2.49 (*P*=.014)^a^	No (*P*>.99)
Maximum estimation			
Cough	No (*P*=.064)	−0.84 (*P*=.011)^a^	Yes (*P*=.026)^a^
Sore throat	No (*P*=.098)	−1.48 (*P*=.111)	No (*P*=.139)
Abdominal pain	No (*P*=.079)	−2.67 (*P*=.042)^a^	No (*P*>.99)

a Significant at *α*=.05

### Time Series Regression

The linear interpolation estimates led to significant time series regression models for all three symptoms. For cough, sore throat, and abdominal pain, the model coefficients were −0.84,−1.32, and −2.49, respectively. Using maximum estimates, sore throat became insignificant, but cough and abdominal pain remained significant with coefficients of −0.84 and −2.67, respectively (Table 1).

### Granger Causality

Linear interpolation estimation led to Granger causality (the number of searches can help predict number of visits) for cough and sore throat, but not abdominal pain. The maximum estimation also led to Granger causality for cough, but not for sore throat or abdominal pain (Table 1).

## Discussion

### Principal Findings and Comparison With Previous Works

For all three acute symptomatic concerns studied, after adjusting for the ~35.5 million person population increase in the United States between 2004 and 2019, the yearly internet search volume in the United States increased by 2- to 5-fold over that 15-year period. During that same period, the number of people seeking outpatient care for that same primary complaint significantly decreased by ~40% for two of the three symptoms and remained unchanged for the third.

For cough, there was no significant cross correlation, but there was significant time series regression and Granger causality when using maximum estimation. Therefore, while internet searches and ambulatory visits did not quite change together over time significantly (*P*=.064), there is still evidence that an increase in internet searches for cough can predict a decline in ambulatory visits for that symptom.

For sore throat, cross correlation, time series regression, and Granger causality were all insignificant for the maximum estimation method. This indicates that there is not enough evidence using this data that internet searches for sore throat correlate with or predict ambulatory visits for the same concern.

Finally, for abdominal pain, time series regression was significant, but cross correlation and Granger causality were not. From this, it can be said that there may be a weak association between internet searches and ambulatory visits, especially when considering that the cross correlation was close to significance (*P*=.079), but there is not evidence presented to say definitively that one predicts the other.

Because of the discrepancy between the “eyeball test” that shows internet searches for a symptom increasing over the same time period that ambulatory visits for that symptom are decreasing, and the statistical analysis that had mixed results, it seems evident that there are other variables interacting that have not been taken into account.

It is still clear that internet search volume for these common health symptoms has increased over time from 2004 to 2019, which is not surprising in and of itself. Prior research has shown that a higher percentage of people are turning to the internet first when they have a health question because it is viewed as accessible, direct, effective, and quick [9]. They often view it as a way to save time and money by not having to see a physician [9]. The concurrent decrease in ambulatory visits, at least for cough and sore throat, seem to indicate that these searches are reassuring the general public and not driving an increase in seeking outpatient care, but the analysis done here does not quite prove such a straightforward picture. Possibly as evidence of this, internet searches for stomach pain increase nearly 5-fold, but the population-adjusted number of outpatient visits for that complaint remained stable.

One alternative explanation is that the inverse trends are not being driven by reassurance provided by internet searches, but by necessity to overcome financial and accessibility barriers. In other words, ambulatory visits are not decreasing because people are finding satisfactory answers online, but instead because they do not have a practical ability to see a doctor anyway. In fact, in 2019, 33% of respondents of a Gallup survey said that in the last year, they or a family member of theirs put off medical treatment because of the cost associated with it [18]. People with this barrier, or a similar barrier like insurance coverage or access to a close care center, may be using the internet because it is the only option they have. In this case, we would expect to see the trend seen here, but not necessarily with statistical analysis showing a direct predictive relationship between internet searches and clinical visits.

Overall, there is an undeniable inverse trend in internet searches and ambulatory visits recorded by NAMCS and NHAMCS data, at least for cough and sore throat. However, the statistical analysis indicates that, while there is some evidence that searches themselves are impacting ambulatory visits, possibly through patient reassurance, the entire story is not captured here. It is likely that there are other influencing factors that have not been accounted for and could feasibly play a role.

### Future Directions

Just as the last two decades saw rapid change in internet tools and use, the current search habits of individuals are in constant flux. Today, the digital landscape is being populated more and more with artificial intelligence (AI) tools, like large language models (LLM) or AI “chatbots,” that are already being widely used by patients to find health care information [12,19]. According to one survey from 2024, 64% of adults have used AI and 17% claim to use AI chatbots at least once a month to find health information [20]. The ability of LLMs to understand human queries, access a huge body of information, then synthesize findings into accurate natural language responses in easy-to-understand terms allows the delivery of customized content to users [21,22]. Patients are able to find information about conditions and treatments in a way that mimics human interaction, but without worry of physician availability or judgment [23]. The pursuit of agentic AI models that are able to essentially “think ahead” take these already impressive abilities one step further. They would be capable of long-term memory and longer reasoning chains that allow initiative taking instead of just response to user input [24]. It seems likely that LLM generated answers to health queries, which in some studies have already been shown to be of higher quality and show more empathy than physician’s online answers to questions, will drive even greater change in internet search habits of users, and potentially completely transform interactions with the health care system [25].

### Limitations

Google Trends accounts for individual IP addresses, but it is still not possible to prove that each search is due to one individual feeling a symptom for themself. It is entirely possible that they are searching on behalf of another person or simply out of curiosity alone.

Moreover, it is possible that not all clinical outpatient visits were recorded. Though the two surveys seek to encompass office-based, hospital-based office visits, and ED visits, it is subject to sampling bias. Additionally, although certain tables in the NAMCS dataset mention “urgicenters”, urgent care visits seem largely unaccounted for in the data. Telemedicine visits are also not mentioned explicitly in NAMCS and NHAMCS data, leading to ambiguity about their inclusion. Missing NAMCS data for 2017 and NHAMCS data for 2012‐2017 could additionally skew analysis. Another limitation exists in not capturing all search queries made for the related symptoms. Differences in phrasing could lead to exclusion from this Google Trends data.

Seasonal variability is to be expected in both search volume and clinical encounters for all three symptoms. However this cyclical nature should not have any impact in comparing year to year trends.

Finally, coding and delayed care could be altering data in ways that are not apparent. As coding practices change over time, it is possible that NAMCS and NHAMCS data have changed with it.

### Conclusions

The internet as a whole has greatly evolved over the last 20+ years and today is at the beginning of undergoing its greatest change since its inception with the incorporation of LLMs. With that change comes change in how users interact with it, including when it comes to searching for health information. There are motivations for internet searches at every step in the health care visit process, which includes even before deciding to go see a physician at all. As internet search volume for health information has increased exponentially over the last two decades, it had the potential to drive either an increase or a decrease in visits to health care professionals. What has been illustrated here is that, in the case of cough, stomach pain, and sore throat in the United States in 2004‐2019, a 2- to 5-fold increase in internet search volume was associated with either a decrease or no change in outpatient visits. This lends evidence to the theory that the internet has provided a place for individuals, on a population-level, to find reassurance for their symptoms to the point where they feel it is unnecessary to see a health care provider. However, it is likely that there are other influencing factors affecting these inverse trends that have not been fully elucidated by this analysis and more work should be done to highlight these. As the ability and availability of AI continues to grow, the impact of these tools on the use of health care resources must be addressed to offer patients the best possible care.

## Abbreviations

AI: artificial intelligence
ED: emergency department
HINTS: Health Information National Trends Survey
LLM: large language model
NAMCS: National Ambulatory Medical Care Survey
NHAMCS: National Hospital Ambulatory Medical Care Survey
